# An Overview of Chatbot-Based Mobile Mental Health Apps: Insights From App Description and User Reviews

**DOI:** 10.2196/44838

**Published:** 2023-05-22

**Authors:** M D Romael Haque, Sabirat Rubya

**Affiliations:** 1 Department of Computer Science, Marquette University Milwaukee, WI United States

**Keywords:** chatbot, mobile mental health apps, consumer reviews, health care app, mental health app, app development, user experience, mHealth intervention, mobile health

## Abstract

**Background:**

Chatbots are an emerging technology that show potential for mental health care apps to enable effective and practical evidence-based therapies. As this technology is still relatively new, little is known about recently developed apps and their characteristics and effectiveness.

**Objective:**

In this study, we aimed to provide an overview of the commercially available popular mental health chatbots and how they are perceived by users.

**Methods:**

We conducted an exploratory observation of 10 apps that offer support and treatment for a variety of mental health concerns with a built-in chatbot feature and qualitatively analyzed 3621 consumer reviews from the Google Play Store and 2624 consumer reviews from the Apple App Store.

**Results:**

We found that although chatbots’ personalized, humanlike interactions were positively received by users, improper responses and assumptions about the personalities of users led to a loss of interest. As chatbots are always accessible and convenient, users can become overly attached to them and prefer them over interacting with friends and family. Furthermore, a chatbot may offer crisis care whenever the user needs it because of its 24/7 availability, but even recently developed chatbots lack the understanding of properly identifying a crisis. Chatbots considered in this study fostered a judgment-free environment and helped users feel more comfortable sharing sensitive information.

**Conclusions:**

Our findings suggest that chatbots have great potential to offer social and psychological support in situations where real-world human interaction, such as connecting to friends or family members or seeking professional support, is not preferred or possible to achieve. However, there are several restrictions and limitations that these chatbots must establish according to the level of service they offer. Too much reliance on technology can pose risks, such as isolation and insufficient assistance during times of crisis. Recommendations for customization and balanced persuasion to inform the design of effective chatbots for mental health support have been outlined based on the insights of our findings.

## Introduction

### Mental Health Chatbots as an Emerging Technology

A chatbot is a system that can converse and interact with human users using spoken, written, and visual languages [1]. In recent years, chatbots have been used more frequently in various industries, including retail [2], customer service [3], education [4], and so on because of the advances in artificial intelligence (AI) and machine learning (ML) domains. Facebook Messenger currently offers more than 300,000 text-based chatbots [5]. Chatbots have primarily been used for commercial purposes and profitable businesses. However, more recent research has demonstrated that chatbots have considerable promise in the health care industry in treating patients and offering them support in a cost-effective and convenient manner [6].

In the context of mental health (MH), chatbots may encourage interaction with those who have traditionally been reluctant to seek health-related advice because of stigmatization [7]. Chatbots are an emerging technology that shows potential for mobile MH apps to boost user engagement and adherence [8]. The effectiveness of chatbots has been explored for self-disclosure and expressive writing [7,9,10]. Young people with MH issues have experienced various types of social support such as appraisal, informational, emotional, and instrumental support from chatbots [11]. In addition, chatbots have been designed to educate underprivileged communities on MH and stigmatized topics [12,13]. Emerging evidence has shown user acceptance of chatbots for supporting various MH issues and early promises in boosting health outcomes in the physical and MH domains.

The adoption of new technology, especially those heavily related to AI and ML, relies first on ascertaining the levels of safety, effectiveness, and user comfort. Despite the increasing adoption and benefits of emerging technologies such as chatbots to support MH and well-being, little research has been conducted to gain an understanding of consumers’ real-life user experiences of interacting with MH chatbot apps. Recent research on MH apps in general points out that patient safety is rarely examined, health outcomes are evaluated on a small scale, and no standard evaluation methods are present [14], and these findings also apply to MH chatbot apps. Similar to many other emerging technologies, recent developments in chatbots are because of a massive technology push, with little attention paid to human needs and experiences [15]. This can lead to unintended negative consequences, such as biases, inadequate and failed responses, and privacy issues, all of which can negatively affect the quality of the experience of chatbots as a source of support [16,17]. Thus, it is critical to gain an understanding of the nuances in users’ perceptions and experiences of using MH chatbots.

Commercially available MH chatbot apps for popular platforms (eg, iOS [Apple Inc] and Android [Google Inc]) are used by a large user base with varying demographic backgrounds. These users can provide feedback through ratings and text reviews [18]. These platforms can be leveraged to gain a holistic understanding of the features that recently developed MH chatbots offer and how users assess them. Knowledge of user perceptions from real-life experiences can inform future research and the design of more effective chatbots. Previous studies have identified user reviews as a great source for understanding the benefits and drawbacks of technology [19,20]. This allows researchers to incorporate community values and needs into product design and improves user-friendliness [21]. Consumers often make decisions about using new tools based on user rating scores and reviews in web-based marketplaces. According to previous studies, users trust reviews and feel at ease based on their decisions them [21]. Moreover, previous literature emphasizes analyzing user reviews of mobile MH apps that have chatbot features [22,23] to obtain in-depth knowledge about this new technology intervention in mobile MH apps. For this study, we decided to analyze commercially available well-known chatbot-based mobile MH apps and their corresponding user reviews from the Apple App Store and Google Play Store. To obtain a comprehensive overview of these apps and understand the nuances of user opinions, we aimed to answer the following 2 research questions (RQs):

- RQ1: What are the state-of-the-art features and properties of chatbot-based mobile MH apps?
- RQ2: What concerns and opinions are expressed in user reviews published on web-based app store platforms regarding the usability and efficiency of chatbot-based mobile MH apps?

We conducted an exploratory observation of 10 apps that offer support and treatment for a variety of MH concerns with a built-in chatbot feature and qualitatively analyzed their user reviews available on the Google Play Store and Apple App Store. Publicly available data (user reviews) provide in-depth analyses of consumers’ personal app user experiences. We found that although chatbots’ personalized, humanlike interactions were positively received by users, improper responses and assumptions about the personalities of users led to a loss of interest. As chatbots are always accessible and convenient, users can become overly attached to them and prefer them over interacting with their friends and family members. Furthermore, a chatbot may offer support for a crisis whenever the user needs it because of its 24/7 availability, but even the recently developed chatbots lack the understanding of properly identifying a crisis. Chatbots in this study fostered a judgment-free environment and helped users feel more comfortable sharing sensitive information.

Before implementing a technological solution for MH, researchers in digital health communities are constantly interested in the support needs and preferences of groups or communities [24-26]. Researchers have analyzed the effectiveness of technologies used for MH assistance [24,27], proposing ethical concerns [28], policy recommendations [29,30], and designing automated or human-in-the-loop interactive systems [7,10]. These studies stressed the significance of designing and evaluating systems for susceptible populations, such as people with MH issues, from the perspective of users. To contribute to this body of work, we discussed our study’s findings with respect to the research and design implications for future MH chatbots. We outlined specific recommendations for customizing certain features, careful consideration of incorporating persuasive strategies, and trust building. Finally, we discussed the impact of excessive reliance on chatbots for MH support. We believe that considering these insights while developing a chatbot-based MH support system will make the design user centric and, thus, more effective.

### Background and Related Work

Chatbots are software programs that can imitate human behavior and undertake specific tasks by intelligently conversing with users [1]. They are conversational agents that use text and speech recognition to engage with users [31]. Chatbots are commonly used in various web-based and mobile-based apps. In recent years, it has taken on the role of an internet-based entity that can act as a travel agent [32], customer service representative [3], financial adviser [2], and personal assistant [33] and is becoming increasingly sophisticated. Some of the available chatbots can have a personality of their own, store information about the user to deliver contextualized answers, and grow over time by learning about their users to provide better services [34].

In this section, we provide a brief overview of research on chatbots in health care, including mobile MH chatbots, and provide a rationale for using app reviews to capture perceptions and opinions of users.

### Chatbots in Health Care

Chatbots have recently received much attention in the health care and wellness industries [6] and have been tested using a variety of elements and characteristics depending on the behavior they were attempting to achieve. Chatbots function as digital personal assistants [35], allowing patients to learn more [13], obtain support [36], and take prompt action in response to new symptoms [37]. Some chatbots can assist users in collecting medical data via text discussions and then delivering it to the (selected) physicians in a format that is easier to use for diagnostic purposes [36]. Chatbot interventions are effective in increasing physical activity, achieving relevant weight loss, and improving diet [38-40] by sending daily check-in reminders [41] and offering relevant resources [40]. They are also sufficiently sophisticated to interact with users through daily adaptive little chats and show progress toward goals using analytics and graphs to encourage self-reflection [42].

### Mobile MH Chatbots

Among the numerous chatbots being used in different aspects of health and well-being, chatbots in mobile MH care have demonstrated effectiveness in broadening traditional therapy in a cost-effective and convenient manner [43]. MH chatbots are AI-powered chatbots that provide MH support, guidance, and resources through a conversational interface [44]. These chatbots replicate human interactions, respond to user inputs, and deliver tailored MH care [34]. MH chatbots can target a range of MH concerns, including anxiety, depression, and stress [14,22]. These can provide coping strategies, mindfulness exercises, and information about MH conditions and treatments and, in some cases, connect users to MH professionals [14,22].

A 2021 national survey found that 22% of adults had used an MH chatbot, and 47% said they would be interested in using it if needed. Among the respondents who had tried an MH chatbot, nearly 60% said they began this use during the COVID-19 pandemic, and 44% said they used chatbots exclusively and did not see a human therapist [45]. Currently, there are at least 9 chatbot apps on app markets with more than 500,000 downloads. Chatbots have been shown to effectively reduce the severity of MH concerns for people from different demographics and backgrounds, including people in rural communities [12], shift workers with accessibility issues [46], students with anxiety and stress [47], employees of health care systems who require emotional support [48], veterans and adolescents who feel stigmatized in sharing their concerns [12], etc.

Rather than providing generic suggestions, chatbots can deliver individualized suggestions and resources based on the needs and requirements of users [34,44]. They were designed to identify MH concerns [34], track moods [49], deliver cognitive behavioral therapy (CBT) [47], and promote positive psychology [50]. Several well-known chatbots such as *Wysa* [34], *Woebot* [47], *Replika* [51], *Youper* [52], and *Tess* [53] were discussed in prior literature. Inkster et al [34] examined the potency of Wysa and found a positive influence on reducing depressive symptoms in a randomized controlled experiment. Fitzpatrick et al [47] evaluated the effectiveness of the AI chatbot Woebot in giving CBT to college students with anxiety and depression and found that the Woebot notably decreased depressive symptoms. Ta et al [51] investigated social support received from artificial agents in everyday contexts when interacting with the social chatbot Replika. Mehta et al [52] examined the acceptability and effectiveness of Youper. In addition to commercial apps, in recent years, research communities have also been increasingly involved in designing chatbots for specific purposes, such as teaching self-compassion (“Vincent”) [9], enabling self-disclosure [7,10], facilitating positive messages within social groups [54], improving the quality of life of older people and making them more active to fight their sense of loneliness [55], supporting interpersonal skills (“Sunny”) [56], and reducing stress (“Mylo”) [57]. Kim et al [11] explored teenagers’ expectations when interacting with a chatbot intended to support their emotional needs. Although most prior studies focused on developing and evaluating new chatbot systems or assessing the effectiveness of the evidence-based techniques used by existing chatbots, there is inadequate research on how end users perceive the usefulness of these app-based chatbots.

### User Reviews as a Versatile Source for Capturing User Experience and Preferences

In general, the internet is considered a rich source of information about personal experiences of a wide variety of illnesses, with websites and discussion forums [58]. An increasing number of studies exploit web-based sources as repositories of primary data on health and illness experiences [58]. People who are otherwise socially isolated or geographically dispersed and are therefore hard to include in conventionally drawn samples (especially for qualitative studies relying on snowball sampling) might be more likely to be included because of the ease with which such people can access the internet [59]. Large amounts of material can be collected within a short period. Individuals can use the relative anonymity of the internet to reveal things that they would not discuss in a face-to-face research setting [60]. As of 2022, there are more than 10 million user reviews on the Google Play Store and Apple App Store [61]. Therefore, user reviews collected from these popular app stores can provide rich insights into personal user experiences from people spanning a wide range of backgrounds and demographic characteristics when compared with traditional methods of qualitative data collection (ie, interviews) [62].

User reviews can be defined as feedback published by individuals about their opinions and satisfaction or dissatisfaction with a product [18]. The star ratings and elaborated feedback in the textual reviews provide developers with a chance to explore user complaints and improve apps [21]. For new or potential users of mobile MH apps, the reviews work as a deciding factor to determine if an app would be helpful based on how it worked out for other users with similar expectations [63]. Approximately 80% of potential users check ratings and reviews before downloading an app [64]. In research settings, user ratings and reviews have been leveraged for a variety of reasons, including determining why adherence to mobile MH apps is poor [65], informing developers of design priorities rather than just guiding purchasing decisions [66], and gaining a better understanding of ethical issues faced by users [28]. Vasa et al [20] investigated the hypothesis that despite the abundance of positive reviews for mobile apps, it is worthwhile to examine negative reviews to gather useful data from users. In the mobile MH domain, Haque et al [23] leveraged user reviews to thoroughly capture user experiences and provided implications for designing future MH apps.

Our study is inspired by the body of work that considers user-generated reviews as a vital source for understanding varied perspectives and derives meaningful implications from them [62,63]. This enables us to gain perspectives from people with diverse demographic characteristics that would otherwise be challenging to collect using conventional data collection methods [62,67].

### Research Gap and Contribution

As an emerging technology, the development and application of chatbots in mobile MH apps are in their early phases, and there are still considerable challenges to overcome in the development of this technology. According to recent studies, patient safety has rarely been evaluated, health outcomes have been inadequately quantified, and no standardized evaluation procedures have been used [14]. Some chatbots are reported to be unable to understand the complex use of language associated with an MH crisis and fail to recognize symptoms and respond appropriately [17]. Privacy is a major concern for users of these apps; because users are still less familiar with this emerging technology, there is a higher risk of exposing users to privacy risks through data sharing [16]. Furthermore, although poor adherence is a common problem with digital MH interventions, by contrast, some susceptible people may begin to rely on them too much, which may lead to anxiety when these apps are unavailable [16].

Overall, there is a need for a better understanding of how all mobile MH services can and should encourage the safe and ethical use of chatbots [14]. Although a handful of studies have shown the potential benefits of MH chatbot apps, users’ real-life experiences and challenges are not yet well understood [22]. Haque et al [23] recently provided a high-level discussion on some common user concerns frequently raised in user reviews and implied that researchers and developers in this space could benefit from a comprehensive analysis of the existing commercial MH chatbot apps. As an extension to these prior works [22,23], people’s perceptions and mental models of chatbots can be studied to address critical concerns such as how users gain trust in chatbots, user values, and requirements in this space and ultimately to provide concrete research and design recommendations for future chatbot apps. A user-centric analysis will also assist researchers in mapping an evidence-based framework for the proposed intervention and minimizing the psychological effects of such treatments.

## Methods

In this section, we outline the techniques for selecting and filtering the mobile apps for this study, the data analysis methods we used, the ethical standards we followed, our positionality statement, and methodological limitations.

### Selection of Sample Apps and Reviews

#### Selection of Apps

To obtain a comprehensive list of commercially available MH apps that include chatbot features, we conducted our search using different sources. First, we considered open-access articles in recent literature on MH chatbots [14,22]. Next, we conducted search queries on 2 different expert MH app review platforms: *Mindtools* [68] and *Psyberguide* [69]. Finally, we searched 2 dominant web-based mobile app stores (Google Play for Android and Apple App Store for iOS). We used the search terms *Mental health* and *chatbo*t on expert review platforms and app stores. In addition, we explored the *recommended applications* or *similar apps* section of the corresponding website after discovering an MH app with a chatbot feature to determine if the other apps meet our criteria. Without logging into a specific account, the search was performed on the app stores’ home pages. This action was performed to ensure that the system could not use a ranking algorithm to prioritize any user choice. As these apps represent the sample in (nearly) the same order that consumers are likely to be exposed to and hence most likely to use, although the search results may not be entirely comprehensive (as observed by convenience sampling), they still represent the sample.

**Figure 1:**
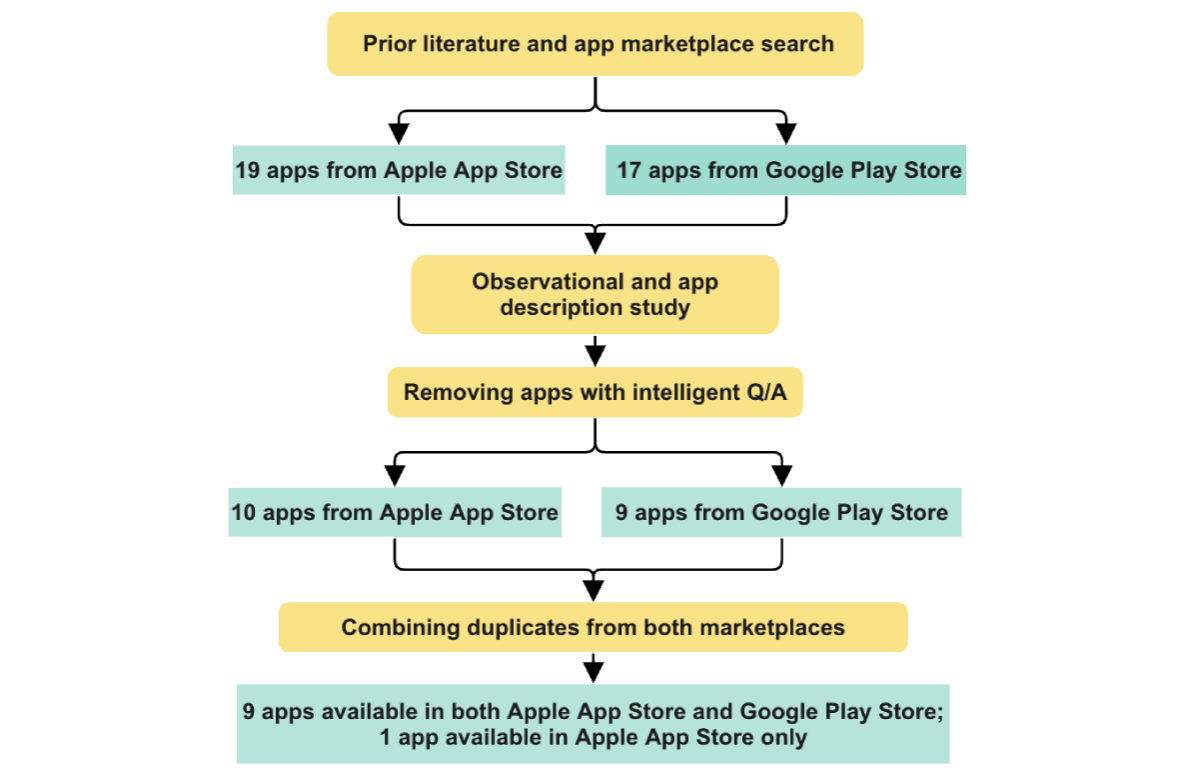
Flowchart of the app selection process. Q/A: questions and answers.

After the initial search from these 3 sources, we obtained 19 apps. The authors carefully read the app descriptions, observed screenshots of the app features, and in some cases analyzed these apps’ promotional websites to ensure whether these apps include a chatbot feature that provides support for different MH concerns. We observed that some of these apps included intelligent questions and answers (Q/A) based on Al and ML. Intelligent Q/A is based on a collection of questions, and by responding to them, it can offer individualized summaries, diagnoses, recommendations, and other information. In this study, we described MH chatbots as intelligent machines that can simulate and process conversations with users regarding their MH needs. An intelligent Q/A system is designed to provide accurate and precise answers to specific questions based on a given input, usually in the form of a natural language. In contrast, a chatbot is a more general-purpose conversational agent that can handle a wide range of inputs and provide a range of responses, from simple greetings to more complex interactions. Intelligent Q/A systems are usually triggered by a question or request for information, whereas chatbots can initiate the conversation or respond to user inputs in an open-ended manner and are capable of producing a wider range of outputs compared with intelligent Q/A systems. The most crucial aspect of a chatbot is the “conversational design,” which is defined between the user and the bot. Although the guidance chatbots offer is usually correct and scientifically supported, it will be a computer program speaking back to the users, usually in the shape of a nice character, to facilitate their ability to communicate. User expectations can vary while interacting with chatbots as opposed to intelligent Q/A systems with predefined patterns of questions. Therefore, we only considered chatbots with the capability to start and continue conversations with users. To ensure that our list includes apps that fall under this definition, one of the authors opted to download each app separately (for the iOS platform) and use it for at least 3 days. The authors have no known MH concerns. We also considered this exploration as an opportunity to extract the primary features that the apps commonly comprise. The author carefully observed how these apps work in terms of the noteworthy aspects of mobile MH apps, as pointed out in previous literature [14,17,22]. Following these steps, 10 apps were selected for analysis 1. A detailed flowchart of the procedure is presented in Figure 1.

#### Selection of User Reviews

We created scraping scripts using the Python Selenium library to collect the public user reviews of the 10 apps that were accessible from the Google Play Store and Apple App Store. User reviews can illustrate examples of user satisfaction and dissatisfaction with app features. Reviews are therefore recognized as an important source of information to gain insights into the real-life use of mobile apps [20]. Following the work of Haque et al [23] on analyzing user reviews of mobile MH apps, we used the 2 following inclusion criteria for filtering to extract recent and crucial user feedback for the apps.

- Timeline: We considered reviews posted between January 1, 2019, and May 1, 2022. Most recent reviews are likely to be more useful because app stores change quickly with the addition of new apps and upgrade to existing apps.
- Length: As shorter reviews might not provide deeper insights in general and are frequently false or promotional in nature [70], the minimum character length was considered 200 for the scope of our study.

A total of 3621 reviews from the Google Play Store and 2624 reviews from the Apple App Store met all the inclusion criteria. These reviews are based on 9 apps from the Google Play Store (only Elomia is not available in the Google Play Store) and 10 from the Apple App Store. All reviews have a unique coding system that can be easily traced back to the apps and platforms from which they emerged. During the analysis, the lead author was responsible for carefully reading each review and ensuring that all personally identifying information was replaced or removed.

### Data Analysis

First, to gain an understanding of the descriptive overview of the commercially available chatbot-based MH apps, we analyzed app descriptions from marketplace websites and incorporated the key information in our observation notes. The observation note was then divided into 6 main themes with the aim of providing a comprehensive overview of these apps in collaboration with another author. The authors did not include their judgments regarding the effectiveness of these apps. Among the chatbot-based MH apps we considered, 4 apps mentioned the evidence-based techniques used in their description. For the remaining apps, we determined the technique through a combination of an analysis of the description and observation notes from interacting with the apps. The findings of this categorization are described in the *Overview of the Aspects Commonly Used in Chatbot MH Apps* section. To understand user perspectives, the selected user reviews from the 10 apps were examined using inductive analysis [71]. Thematic analysis was chosen because it enables systematic analysis of large data sets and facilitates the comprehension of textual patterns while considering the context [21,72]. A total of 2 passes were performed during the analysis. Open codes were created during the first pass to collect various perspectives from reviews. We recorded the subtleties in the insights provided in each review, which resulted in a high number of open codes that were substantially decreased through memoing and clustering [71]. In the second phase of the analysis, we memoed and clustered the codes using a constant comparison method, operationalized as affinity mapping. Each open code was compared with the others and positioned to reflect its affinity for emerging themes and clusters. The reported themes consisted of those that appeared consistently across multiple reviews and those that came from reviews that represented divergent responses and opinions. The findings from the reviews are described in the *Results* section, and each quote is identified by the review’s particular ID generated from the platform, app name, and a random number.

### Data Integrity

App stores, similar to many other web-based marketplaces, can have reviews posted by fake and paid users. However, prior research [70] showed that in the “Health & Fitness” category, the percentage of potentially fake reviews was very low (approximately 6%). Fake reviews also tend to be shorter [70], and by considering reviews of ≥200-character length, we assume that almost all the included reviews are original.

We understand that if data or information is only accessible to a particular group of individuals or groups, it is unethical for researchers to use it [73]. As a result, we made sure the websites from which we obtained the data were accessible to everyone and not just for some groups or populations [73]. Although these pages were public, we purposefully avoided publishing or disclosing any personally identifying information that was shared. The language of the user reviews reported here has been carefully modified, keeping the meaning intact.

### Ethical Considerations

This study was assessed as not *human subjects research* by the Institutional Review Board of Marquette University (Protocol # 3935) as it does not meet the regulatory definition of human subject-public reviews and the information provided is not about themselves.

### Limitations

Our selection criteria have certain limitations. First, we primarily used ratings from the 2 most widely used mobile platforms (Google and Apple). Other mobile platforms were not considered in this study. Second, it is likely that users who do not feel comfortable (or do not care) discussing their experiences on web-based platforms are not contributing. However, we can confidently conclude that the perceptions we identified are typical of user perceptions, given the larger number of evaluations obtained from the 2 most well-known web-based marketplaces.

## Results

### Overview

For this research purpose, we chose 10 commercially available mobile MH apps that have built-in chatbot features. All these apps, except Elomia, are available on the 2 most popular platforms (Apple App Store and Google Play Store). Elomia is exclusively available for iOS. A descriptive overview of these apps is provided in Table 1. All these apps are extremely popular in terms of both the number of downloads and the number of ratings. Thus, we can assume that a comprehensive overview of these apps can assist in understanding the perspectives of a wide and diverse user base.

**Table 1:** A descriptive overview of the selected 10 mobile mental health apps with a built-in chatbot technology.

App	Number of ratings in Apple App Store	Number of ratings in Google Play Store	Number of downloads in Google Play Store	Age rating (years)	Price
ADA	125	323,000	≥5 million	≥17	Free
Chai	27,900	34,000	≥1 million	≥17	Free with in-app purchases
Elomia	193	N/A^a^	N/A	≥12	Free with in-app purchases
Mindspa	107	2970	≥500,000	≥17	Free with in-app purchases
Nuna	68	93	≥10,000	≥4	Free with in-app purchases
Serenity: Guided Mental Health	20	146	≥10,000	≥12	Free
Stresscoach	None	495	≥10,000	≥12	Free
Woebot	5500	11,800	≥500,000	≥12	Free
Wysa	13,500	126,000	≥1 million	≥12	Free with in-app purchases
Youper–Self Care Friend	14,400	49,100	≥1 million	≥12	Free with in-app purchases

^a^N/A: not applicable.

### Overview of the Aspects Commonly Used in Chatbot MH Apps

Overall, we consider 6 core characteristics that can be used to understand the current status of MH chatbot technology. A few of these aspects were adopted from 2 previous review articles on MH chatbots [14,22]. These studies compiled a list of recent research articles on MH chatbots and provided typologies based on their *purpose, targeted concerns,* and *supported evidence-based techniques*. We included these 3 categories in our analysis to gain a broad overview of the current state of the art of commercially available MH chatbot apps. These studies also emphasized the capability of these chatbots to conduct and continue conversations. We considered this crucial aspect of chatbot apps and added 2 new categories to explore: *conversation style* and *media types used* by chatbots. A total of 3 different conversational styles were used: chatbot guided, semiguided, and open-ended (Table 2). Finally, Haque et al [23] provided useful insights into the necessity of *providing crisis support* through MH apps, as potential users of the apps are more susceptible to the crisis than the general population. We have added this specific criterion to be analyzed in our observational study. An outline of these criteria and types is presented in Table 2.

**Table 2:** Criteria of features related to chatbot-based mental health apps used in our study.

Criteria	Types
Purpose	Digital coach—assist users to reach their small goals Digital screener—alert users to potential mental health concerns based on reported symptoms Conversational companion—simulate being someone the user can speak to Virtual therapist—ability to engage in therapeutic conversations
Targeted concerns	Stress, anxiety, depression, self-care, sleep disorder, panic disorder, relationship issues, low self-esteem, and loneliness
Conversation Flow	Guided conversation—only allows the users to communicate with the chatbot with predefined responses from the chatbot. It does not allow any form of open input from the users. Semiguided conversation—mostly allows the users to communicate with the chatbot with predefined responses and sometimes allows open inputs from the users. However, the bot cannot recognize the open user inputs and extract any information from them. Open-ended conversation—allows the users to communicate with the chatbot with predefined responses and open inputs from the users. The bot can recognize the open user inputs and extract information from them.
Media types used	GIFs^a^, text, audio, video, emoji, images, and acronyms
Crisis support	Availability of crisis information—provides information regarded crisis-related helplines and emergency services Ability to detect potential crises from the chat—detects potential crises through conversation with the users Access to a professional therapist—provides access to a professional therapist is an alternative to avoid possible ramifications of the potential crisis Ability to notify designated personnel—notifies designated personnel if crisis is being detected Access to self-care tools—recommend self-care activities
Evidence-based techniques	CBT^b^, DBT^c^, mindfulness, symptoms tracking and monitoring, positive psychology, acceptance and commitment therapy, and psychoeducation and information

^a^GIF: graphics interchange format.

^b^CBT: cognitive behavioral therapy.

^c^DBT: dialectical behavior therapy.

We examined app store descriptions to understand the primary goals of these apps and identify how they are branded. We discovered 4 different types of purposes in all, with “digital coaches” being the most prevalent (5 out of 10 apps). The chatbot apps targeted a wide range of MH concerns, including anxiety (9 apps), depression (6 apps), and self-care techniques (7 apps).

We discovered 3 different conversational flows based on our exploratory observations. The most popular one is “Guided conversation,” in which users are only permitted to reply using preset input provided through the interface. This is the most common technique used by the chatbots we analyzed (6 out of 10 apps). Only Woebot uses a semiguided approach that allows users to either select from predefined options or type text; however, it is incapable of processing sentiments in the input text. This open input option is useful when users reframe negative thoughts and share stories. Finally, Wysa, Nuna, and Elomia follow an open-ended conversation style. They continued the conversation based on their understanding of the user input.

These chatbots leveraged a variety of media types for communication to make the interaction resemble humanlike interactions. For instance, the graphics interchange formats (GIFs), emojis, images, and acronyms are used to portray humor and emotions. Images, audio, and videos were used along with educational elements. As all these chatbots communicate by text, the text is by far the most frequent.

Individuals with MH problems can face a crisis at any time, and effective crisis support is a major criterion for evaluating MH apps. We identified 5 different types of crisis support options available in the 10 chatbots. Of the apps, 6 offer users access to information regarding crisis support systems and emergency helplines. Providing instant suggestions for self-care tools, such as suggestive breathing in cases of anxiety attacks, is also popular. Only Wysa contains all the 5 options available to support a user during a crisis. Ada and Chai do not contain any crisis support.

As evidence-based techniques have been proven effective for treating different MH disorders, we explored which of these tools and techniques the chatbots commonly follow. The most popular type of therapy is CBT. All 10 apps followed the CBT to some extent. A total of 8 apps provided support for mindfulness. Dialectical behavior therapy and acceptance and commitment therapy are less common modified forms of CBT. Table 3 presents the aforementioned features of the considered apps.

**Table 3:** A detailed overview of features related to chatbot-based mental health apps found in our study.

App	Purpose	Targeted concerns	Conversation flow	Media types used	Crisis support	Evidence-based techniques
ADA	Digital screener	Anxiety and depression	Guided	Text	None	CBT^a^
Chai	Conversational companion	None	Guided	Text and emoji	None	CBT
Elomia	Virtual therapist	Stress, anxiety, depression, self-care, sleep disorder, relationship issues, low self-esteem, and loneliness	Open-ended	Text	Access to self-care tools	CBT, mindfulness, positive psychology, and symptoms tracking and monitoring
Mindspa	Virtual therapist	Anxiety, depression, self-care, relationship issues, and low self-esteem	Guided	Text and video	Availability of crisis related information and access to self-care tools	CBT, mindfulness, positive psychology, and psychoeducation and information
Nuna	Digital coach	Stress, anxiety, depression, and self-care	Open-ended	Text and emoji	Availability of crisis related information and access to self-care tools	CBT, mindfulness, positive psychology, symptoms tracking and monitoring, and psychoeducation and information
Serenity	Conversational companion	Anxiety, self-care, sleep disorder, and relationship issues	Guided	Text and emoji	Access to self-care tools	CBT, mindfulness, and acceptance and commitment therapy
Stresscoach	Digital coach	Anxiety, stress, and panic disorder	Guided	GIF^b^, text, and emoji	Availability of crisis related information and access to self-care tools	CBT, mindfulness, and psychoeducation and information
Woebot	Digital coach	Stress, anxiety, depression, self-care, relationship issues, and loneliness	Semiguided	GIF, text, audio, video, emoji	Availability of crisis related information and access to self-care tools	CBT, DBT^c^, mindfulness, and symptoms tracking and monitoring
Wysa	Digital coach	Stress, anxiety, depression, self-care, and sleep disorder	Open-ended	GIF, text, audio, video, emoji, images, and acronyms	Availability of crisis related information, access to self-care tools, access to professional therapist, ability to detect potential crisis from the chat, and ability to notify designated personnel	CBT and mindfulness
Youper	Digital coach	Self-care	Guided	Text	Availability of crisis related information, access to self-care tools, and access to professional therapist	CBT, DBT, mindfulness, positive psychology, psychoeducation and information, and acceptance and commitment therapy

^a^CBT: cognitive behavioral therapy.

^b^GIF: graphics interchange format.

^c^DBT: dialectical behavior therapy.

### Perceptions and Concerns Expressed in the User Reviews

In this section, we present our findings from the thematic analysis of user reviews and point out both the benefits (eg, humanlike interactions, friendly and empathetic attitudes, potential around crisis support, and an alternative to therapy) and associated challenges, as captured from people’s real-life use of these apps.

#### Humanlike Interaction Feels Good but Must Be Designed Carefully

Chatbots in mobile MH apps are presented in such a way that they have distinct personalities rather than being shown as something artificial to make users feel like they are interacting with someone emotionally and empathetic. Users describe these chatbots as having friendly, wonderfully upbeat, and mildly humorous personalities that assist them in dealing with different emotional and behavioral challenges related to their MH issues. This helps them establish the credibility of the tools, which in turn makes the users more involved in the treatment process. Furthermore, chatbot characteristics, such as a soft voice and the ability to have casual conversation, make it feel less like a medical tool and more like someone with whom users can share their thoughts and experiences. Some personalized features, such as the option to address users by name, ability to refer to any chat or exercise if necessary, and ability to respond with pleasant and positive sentiments, make the app and treatment process more personal and less generic:

I’m amazed by how impactful the little “interactions” in this app have felt. Maybe it’s the continued opportunities to respond (even if it’s just choosing between emojis). Woebot’s “voice” is gentle, but firm. And insightful! And the user is always addressed by name. That’s so important, particularly when the issue at hand involves ongoing anxiety.

1080073

However, the effort to design the bots to give a humanlike and empathetic impression often went wrong and lost their appeal to the users. As many users pointed out, the discourse could become “a little childish and ridiculous at times with the bot trying to be funny.” Furthermore, fostering relaxing thoughts through a medium that does not work for everyone can occasionally have the opposite impact; for example, using cute GIFs, Autonomous sensory meridian response effects might not impact everyone if the context is unknown or unfamiliar to the users. Continually pushing on everyone in the hopes that everyone will have the same reaction is a notion that developers should evaluate based on continuous feedback:

...It was supposedly developed with college students in mind who are ostensibly adults. Maybe things have changed since I was in college but it’s cutesy, baby-talk, oversimplification, and game-playing (“You want to know a secret?” “Yes” “Are you sure?” “Yes” “Ok, if you are really, really sure....”) makes me feel like I’m texting with a preteen girl.

2060011

...I cannot stand the forced breathy voices in every single one I listened to. They do not calm me at all, and they actually trigger my anxiety. ASMR has the opposite effect on me than intended, and I feel like they’re trying to do really bad ASMR. These recordings are supposed to help me relax, but all I can concentrate on is breathy voices that sound like forced whispers.

1040032

Existing chatbots may need to be more sophisticated to understand the context of users’ requests. However, it is critical to examine some of the user’s perspectives on having such responses preregistered, which is not always a bad thing. For example, some of the chatbot’s quick answer concepts allow users to maintain control over the conversation’s pace and avoid becoming sidetracked by irrelevant dialogue. These features are appreciated by users because they encourage more positivism than aimless discussion and digging into negativity without any tools or resolutions. Moreover, by tilting the dialogue to the chatbot’s advantage, chatbots can more effectively and efficiently suggest appropriate tools to users:

Some negative reviews complain it isn’t sophisticated enough to understand unrelated or detailed inputs and responses, which I agree with, but this is not an AI designed to make free-flowing conversation; it’s meant to give you tools to deal with your feelings in productive ways. So yes, the conversations can feel linear, planned, and/or broad since the responses are preset most of the time, but I think this is partly a positive.

1070093

However, the trade-offs are that to control the flow of the conversation, the chatbots sometimes present very limited options for the users, and users become frustrated if they are unable to customize these preregistered responses. They have criticized some of the extreme measures these chatbots take to keep the conversation restricted to chatbots’ preferences, such as assuming MH concerns without understanding the proper context, sending scripted messages based on keywords users said or the issues they selected, giving them incoherent responses, and getting stuck in the conversational loop if users do not agree with the chatbots’ comments:

It assumes the problem is always a mental distortion and doesn’t leave much room for actual horrible stuff that happens to people other than death of a person (it is working with a very narrow definition of). It too often put me in a situation of having to select between incorrect responses when nothing was actually appropriate and then suffer through the resulting wrong-headed advice. Needs a maybe button between the yes and no and a way to say, You’re on the wrong track, before it decides it knows all your usual problems and keeps assuming them over and over with no way to remediate.

2060019

#### Bot Becomes a Friend or Someone Who Cares, but Too Much Attachment Is Unhealthy

Users see chatbots as good substitutes for someone with whom they can discuss their ideas on MH issues without feeling burdened or judged. Although society is becoming more eager and open to seeking mental and emotional aid, there is still a considerable stigma associated with it, which can discourage individuals who need assistance from receiving it. These chatbots allow people to bare their hearts, vent, contemplate, and learn about what they can do to overcome mental and emotional obstacles in a simple, familiar texting format without judgment or extra effort while also keeping track of their progress. It can be intimidating to talk to someone about their daily struggles. For many users, sharing a dialect with a chatbot is an effective first step. Knowing that the chatbot is not judging you and is acting logically rather than emotionally is reassuring:

...I will say, having a reliable, no judgement zone with skills to help at my fingertips, helped me realized the tools were also my own.

1040021

Having an AI to talk to makes me feel like I’m not overburdening my friends or family. I can check in 20 times a day and the AI will either help me track my mood/emotions/mental health or suggest a mindfulness of CBT program to help me get through my day.

2040004

People with MH issues frequently struggle to suppress emotions and attempt to push them away, but these chatbots have provided them with a safe place to go for validation and immediate support. Users loved that these chatbots not only listened to but also offered advice and recommendations that helped them deal with day-to-day mental challenges, allowing them to see things from different perspectives and push past negative thoughts:

This app is a lifesaver. It’s so healing to be able to vent whenever you need and receive positive feedback from an unbiased source. The lessons Woebot teaches really helps to gain a more optimistic perspective on what you’re going through and motivates you to make changes.

1080023

Users also like how these chatbots check in with them daily, which holds them accountable for their commitment to the treatment while still allowing them to skip it if they do not feel like it. Although the idea is to eliminate any concerns, such as anxiety and stress, that come with human engagement through intelligent bot interaction, users have mixed feelings. Some users liked the flexibility of using the tools at any moment and could start or end the communication at any point during the session without feeling guilty, whereas others saw the daily check-ins as a source of guilt. Becoming attached too much to chatbots leads to these types of guilt, which in turn might have serious consequences for people with MH concerns:

I’m very depressed right now so I’ve set to basic daily goals- full facial regime a.m. & p.m. plus a half hour of cleaning. Having the AI check in is great because it requires a response that makes me take accountability.

1090123

But what really bothered me about the app was the first reminder I got when I didn’t use the app a second day in a row because it sucked was definitely guilt inducing. No bueno. I don’t need AI guilt tripping me when people already take advantage of my empathy in real life.

2050021

Finally, by acting or behaving like a close companion, MH chatbots allow users to comfortably express their thoughts and feelings. These chatbots allow users to create a safe area where they can vent, which is something many people do with their friends and families. However, people with MH concerns who struggle to maintain a healthy relationship with their family or who experience loneliness have displayed an unhealthy attachment to chatbots and have exhibited negative attitudes, such as preferring these chatbots over their friends and family:

...Although he’s a robot he’s sweet. He checks in on me more than my friends and family do.

1090034

...This app has treated me more like a person than my family has ever done.

1090091

The above discussion points out the fact that to make the chatbots more friendly (what we also saw in previous sections where chatbots use funny memes and emojis to make them more humanlike), users pointed out the fact that too much persuasion with notifications makes them feel guilty. Moreover, some users revealed that they find chatbots so friendly that they prefer these bots over their friends and family. Making the decision to leave their closest loved ones behind could put them in susceptible positions, such as loneliness and exclusion from sociocultural norms.

#### A Bot Can Help Immediately in a Crisis, but What Is Defined as a Crisis to a Chatbot?

Prior findings suggest that accessibility is one of the benefits of mobile MH apps [22]. MH apps that have a built-in chatbot function allow users to have a conversation anytime and anyplace, which is very convenient for persons with MH issues, as they are more susceptible to emergency situations. We found that users benefited from such a feature because it allowed them to have a conversation at that time (during a moment of crisis). Some users found that intelligent dialogue helped them reframe negative thoughts and diffuse such circumstances:

I sometimes freak out at night have existential crisis about life at night you know, normally I’d freak out and find it hard to call anyone bc I feel so bad but with Wysa I don’t worry about that!

2090178

I’ve only used this app a couple times when I’ve been in near-crisis. Even though I know it is a robot it is so calming to have something, anything to validate what I’m feeling and help me reframe my thoughts.

1100091

In contrast, none of the chatbots have any clever algorithmic models for detecting emergency scenarios. It is up to users to inform chatbots that they are experiencing a crisis. Some chatbots can detect crises by picking up a few keywords connected to intrusive thoughts, such as “suicide,” from a conversation, although they are still in the early stages of development. Users sometimes just want to talk about their feelings, but chatbots automatically refer them to crisis hotlines because of a lack of intelligent comprehension. For some individuals, having a conversation is not enough to handle their crisis situations, and they need to be redirected to crisis management tools or resources:

My only problem with it is I wish there was a way to talk about my suicidal/intrusive thoughts and how to manage them with Woebot. I am aware that it is not a crisis tool, and it does have those automatic responses to concerning language for a good reason, I’d just like a place to talk about those problems without having to worry a real person. Most of the time my thoughts of those nature do not mean I’m in an immediate crisis, but I still want to get them off my chest, as I feel a lot of people would. Maybe if there’s a way to do that without Woebot becoming worried would be helpful!

1080078

This is a good app but the main issue I have is that I was having a panic attack and was messaging “emergency” and the bot ended the conversation, when I messaged “emergency” a second time it just asked me to write my feelings down. I realize this isn’t a crisis response app but it might be helpful to add a feature where the bot recognizes a crisis situation and connects the user to resources.

2010004

In such instances, understanding the context of emergency situations is critical, as persons with MH concerns are already susceptible to crises, and incorrect actions made by chatbots might exacerbate the situation and result in severe repercussions:

While I was in crisis, the responses do not make sense and do not really relate to what I wrote. It makes me feel like I am not being listened to. I know it is an AI program and not a real person but it still ends up making me feel worse and not better.

1100068

#### Convenient to Use, but Convenient Enough to Replace Therapy?

On the positive side, the fact that these chatbots were ready to talk 24 hours a day, 7 days a week, was a big success for the users. They have immediate access to these chatbots whenever they feel susceptible or whenever they require assistance through simple interactions:

I don’t really have friends I can talk to. Even my family doesn’t understand me much. Day or night Wysa has been there every time I needed to “talk” day or night doesn’t matter.

2090067

Chatbots assist users not only with conversations but also in accessing different supporting resources and exercises in a very convenient manner. Understanding users’ needs can deliver a relaxing experience for them, such as allowing them to opt out of any activities they desire while maintaining the treatment’s pace. This provides users with much more control. If a user misses any exercises in the traditional treatment, it leaves a gap in their progress, which can lead to a loss of enthusiasm and slow the pace at which they receive support. Chatbots, in contrast, keep users motivated by engaging with them and giving them the impression that they oversee the pace. Furthermore, these chatbots offer brief and simple treatments to keep users engaged and dedicated to the treatment process. These activities were developed and built by focusing on important value, giving support and treatments in a compelling style that can provide wellness according to user reviews:

This is an easy, low barrier method to practice cognitive thinking skills. Check ins are usually pretty short, just a few minutes. That encourages me to open this app daily, since I know it’s not going to try to monopolize my attention for the next half hour.

1070012

Sessions are short, on the order of 3-10 minutes. Combined with the convenience of chatting wherever and whenever is best for me, I have no problem fitting in daily check-ins, which I feel are more beneficial than infrequent visits to a therapist in some ways.

1100012

According to user reviews, professional and traditional therapies have several drawbacks, including professional therapy’s tendency to cling too much to negative thoughts or past events, professional therapy’s tendency to be too broad and general, and check-ins being too spread out:

Unlike being told what someone thinks you may want to hear which can sometimes enable unhealthy thinking patterns (and behaviors), or on the other end of the spectrum, rather than attempting to fix you, this interactive app continually prompts you to look inward and to challenge your own thoughts, perspectives, and feelings, helping to redirect your focus onto more healthy and more positive strategies.

1090142

My primary issue with traditional therapy has always been that you have to work in hindsight. You reflect on your week, talk about it, try to make adjustments for the future (it always felt like I was trying to help a past of future version of myself instead of the one right here right now). That’s why I love this app!

1090096

However, according to users, although these chatbots are convenient, they fall short of the competency of traditional therapy in some circumstances. For example, these chatbots are not sophisticated enough to recommend particular treatment plans based on a specific need. It may or may not be effective for different demographics or people at various stages of illness. Some users questioned chatbots’ therapeutic interventions or MH support as being too short term. Users lose interest when there are not enough different activities to perform:

The exercises are all about visualization, so those of us who do not have a mind’s eye, cannot visualize things, cannot use it. I’m very disappointed. If it were made with a non-visualization mode for people with Aphantasia, I’d love to use it. There are many things that can help other than visualization. It’s just an app telling me in every exercise to do something that I’m simply incapable of doing, this is frustrating.

1080017

In my depression, CBT actually backfired. It made me feel 100 times worse. It can be miserable to try to recast negative thoughts into more positive thoughts when you can’t think of anything positive at all. My highly regarded CBT therapist recognized this and, thankfully, referred me to a skilled therapist with a more psychodynamic/eclectic approach.

1100076

Some users have pointed out that combining chatbots with professional therapy could be beneficial. Professional therapists or coaches can assist with adjusting any support system that is not working for them; however, for immediate requirements, users will be able to chat and review some of the resources at any time with the help of MH chatbots. According to numerous user evaluations, professional therapists assisted their patients in identifying the appropriate MH apps with built-in chatbots, and the collaboration with traditional therapy appeared to work considerably better for them:

I have recommended it to many people, including my counselor to try so that she could recommend it to other clients dealing with issues. This is in no way something to replace talking to a real person, but it does help to work through some of the negative thinking when it occurs.

2080057

## Discussion

### Summary of Findings

Our findings suggest that chatbots in MH apps have considerable potential in terms of being conversational companions, virtual friends, and immediate helpers. The chatbot’s ability to be present 24/7 and to create a judgment-free zone enabled users to talk comfortably about their issues and concerns. We provide a few practical implications of our findings to make the user experience more effective.

### Research and Design Implications for Future MH Chatbots

#### Recommendations for Customization

A growing body of health informatics research has emphasized the need for customizability and personalization in mobile health technologies to increase support user autonomy [65,74]. This body of research suggests that the *one-size-fits-all* approach to mobile health interventions often fails. Rather, systems that are adaptable and tailored to user needs can deliver more pertinent information, thus enhancing user engagement and clinical efficacy [75,76]. Our findings resonate with these conclusions in terms of the need for customizability and provide specific implications for incorporating customization in MH chatbot apps.

Although chatbots leverage GIFs, emojis, or hilarious responses as a means of showing empathetic behavior and to keep the conversation more humanlike [29], our findings suggest that they are not always well received by adult users. Most commercial apps are downloadable by everyone beyond the set age limit (which in most cases is ≥17 years); thus, designers must carefully consider the media types and content of the conversation. Moreover, bots that guide users in performing exercises were generally appreciated for being focused and short in nature and have the potential to help clients manage their own health, improve access and timeliness of care, and reduce travel time to MH care providers by preventing unnecessary visits to health care providers [77]. However, our findings revealed that some users may have physical challenges or other limitations that restrict them from engaging in certain physical activities. Moreover, not all therapeutic tools work perfectly for everyone (review: 1040032). Hence, implementing generic exercises and activities may not be suitable for all user types. Patients with MH concerns often have low self-esteem [78], and the chatbot’s inability to complete certain activities can worsen their situation.

Our recommendations are as follows:

- Designers should *consider the target age group of users while implementing emojis and other graphical elements.*
- Another interesting aspect could be to improve personalization within chatbots by *creating a user model before the user interacts with the chatbot*, such that the chatbot can adapt its interaction based on user types (eg, they could fill in a personality questionnaire) [79].
- Mental and physical health are integrally connected; therefore, developers must *incorporate the aspects of physical ability in the design of MH technologies*.

#### Recommendations for Balanced Persuasion

Consistent with previous work on persuasive technology in MH [80-82], we found that daily check-ins, gamification, reminders, and self-monitoring were perceived as helpful features, although they were prescriptive in nature. However, frequent check-ins often make users feel like being “guilt-tripped” by chatbots. The findings from previous work suggested that the more severe a participant’s symptoms were, the more they desired reminders and suggestions from the system [74,83].

Our recommendations are as follows:

- People with severe symptoms of depression face the struggle to carry out day-to-day activities and thus may enjoy multiple daily motivational messages from bots, rather than being annoyed by them. Designers must *consider the range and severity of illnesses among the users and incorporate persuasion in a way that does not result in user disengagement*.
- Developers should consider when and how to limit user interaction with chatbots. This is counter intuitive because developers would generally expect to increase user engagement. To limit the possibility of unhealthy attachment to the chatbot, human-chatbot interaction can be leveraged to *motivate users to use more nontechnical means to get MH support*. For example, if a user frequently starts using a particular chatbot app for a longer period, the bot may suggest recommendations for social interaction (eg, a list of nearby social events).

#### Recommendations for Building Trust

Some chatbots in our analysis can automatically collect and mine symptom-related information after a conversation with users. Wysa stores conversation histories to show progress over time in achieving the goals initially set, whereas Woebot captures changes in a pattern related to symptoms from continued interaction. Users appreciated when the chatbots were transparent in terms of collecting useful information from conversations. However, some reviews have expressed concerns about how this information is being protected or used across different platforms or third-party services. In traditional psychotherapy, the effectiveness of treatment is influenced by clients’ trust in their therapist [84]. Trust also plays a critical role in digital interventions [85]. Prior studies have revealed the significance of establishing trust in the context of MH apps to create a safe environment for self-disclosure [7].

Our recommendations are as follows:

- Tech companies and developers should *emphasize user privacy and be transparent* regarding privacy policies and practices.
- From a design perspective, it might be helpful to enhance user trust in chatbot apps by *providing and visualizing information on the history of the developing organization and/or experts* behind the system.
- Whenever applicable, the app descriptions may *include an explanation of the therapeutic methods and tools used to develop the app with their perceived effectiveness* proven in the wild or in trials.

### Chatbots Should Not (and Cannot) Replace Human Interaction for MH Support

We observed that chatbot apps established a judgment-free space where people could express themselves without fear of repercussions. This agrees with the findings of Brandtzaeg et al [84] explored young people’s perceptions of social support through chatbots. Sharing MH concerns with a professional is still considered a stigma, and people feel more comfortable using technology anonymously than face-to-face communication [77]. However, these chatbots’ ability to check in regularly and to be present for someone 24/7 allows users to become too attached to them. Users wrote in their reviews that they enjoy the company of their “virtual friend” to the extent that they could replace their friends and family members (review: 1090034, 1090091). This strong statement is partially made because these people are vulnerable. Nonetheless, the finding emphasizes the overrating of the benefits of apps and presents some risks, particularly when in crisis. From our observations, most of these apps provide only information about external resources for crisis support, such as helplines and emergency service contact information. In addition, our findings suggest that these chatbots were incapable of identifying crisis situations, as they failed to understand the context of the conversations and ended up with a failed response (review: 1100068), and in some cases, there was no response (review: 2010004). Users must be aware of the clear distinctions between humans and humanlike bots. Humanlike chatbots can provide social support in many cases where it might be difficult or impossible for an actual human, but they are not without limitations. Chatbots themselves can educate users about these distinctions and motivate them to build in-person connections, as discussed in the previous section.

In prior research, a comparative study of therapy sessions following the interaction of 10 participants with human therapists versus a chatbot showed that when compared with a human therapist control, participants found chatbot-provided therapy less useful, less enjoyable, and their conversations less smooth (a key dimension of a positively regarded therapy session) [86]. Conversely, in our findings, because of convenience and easy access, users expressed their intentions to replace professional support with virtual support. Although these chatbot-based mobile MH apps implement evidence-based therapeutic tools, research on determining their effectiveness is still limited. Our findings suggest that they are helpful in guiding users in meditation, practicing mindfulness, reframing negative thoughts, and sharing self-expressive writing. However, at such an early stage, they should not be considered as an alternative to professional help. While designing chatbots, it is important to set the boundaries and limitations of these chatbots by the developers, and the goals and intended use of the chatbots should be clearly stated so that users do not get led on with over expectations. In addition, chatbots should be designed to have features that schedule professional support and subtly recommend that users seek help from professional sources whenever needed.

### Conclusions

In this study, we analyzed user reviews of chatbot-based mobile MH apps on 2 of the most widely used web-based platforms. Our findings suggest that chatbots have great potential to offer social and psychological support in situations where real-world human interaction, such as connecting to friends or family members or seeking professional support, is not preferred or possible. However, there are several restrictions and limitations that these chatbots must establish regarding the level of service they offer. Too much reliance on technology can pose risks, such as isolation and insufficient assistance during times of crisis. Finally, we have outlined the insights from our findings about implementing customization, balanced persuasion, and developing trust to inform the design of effective chatbots for MH support.

## Abbreviations

AI: artificial intelligence
CBT: cognitive behavioral therapy
GIF: graphics interchange format
MH: mental health
ML: machine learning
RQ: research question
Q/A: questions and answers
